# Septins in the Middle—Makers and Breakers of Membrane Contact Sites

**DOI:** 10.1111/jnc.70495

**Published:** 2026-06-12

**Authors:** TrishaJean J. Holt, Elias T. Spiliotis

**Affiliations:** ^1^ Department of Biology University of Virginia Charlottesville Virginia USA; ^2^ Department of Cell Biology University of Virginia School of Medicine Charlottesville Virginia USA

## Abstract

Intracellular communication in neurons requires precise coordination of signals across geometrically complex and highly compartmentalized cellular architectures. Membrane contact sites (MCS)—specialized junctions where two organelles are closely apposed without undergoing fusion—have emerged as key organizational hubs enabling efficient exchange of ions, lipids, and metabolites, yet the cytoskeletal proteins that organize and regulate these junctions remain poorly understood. Here, we review evidence that septins—a conserved family of heteromeric GTP‐binding proteins that assemble into filamentous oligomers and polymers—function as integral components of MCS in neurons and beyond. Septins associate with hyperboloid, hourglass‐shaped membrane curvatures and distinct membrane domains and organelles through polybasic motifs, amphipathic helices, and transmembrane domains. We review how septins establish diffusion barriers at endoplasmic reticulum (ER)‐plasma membrane (PM) contacts in budding yeast and consider evidence that analogous mechanisms operate at dendritic branch points and spine necks in mammalian neurons. We examine septin roles in regulating store‐operated calcium entry at ER‐PM contacts and explore how septins regulate membrane contacts at presynaptic active zones. Additionally, we highlight how septins organize membrane contacts between host organelles and intracellular pathogens, scaffolding autophagic, mitochondrial, and lysosomal membranes for bacterial clearance. Collectively, these findings support the view that septins constitute a versatile and underappreciated class of MCS tethers whose paralog‐ and isoform‐specific complex compositions may confer spatial and functional selectivity for distinct MCS, opening new avenues for understanding organelle connectivity in health and disease.

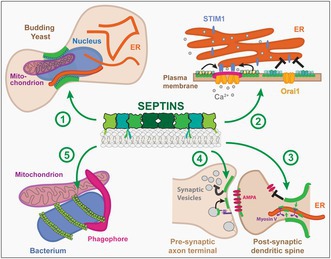

## Graphical abstract

Septins are a family of GTP‐binding proteins that assemble into heteromeric oligomers and polymers, associating with specific membrane domains and organelles according to their subunit composition. Growing evidence places septins at membrane contact sites (MCS) — key hubs for intracellular communication that mediate exchange of ions, lipids, and metabolites between membrane compartments. Here, we review septin functions at MCS across different cell systems, highlighting five roles: (1) formation of diffusion barriers at plasma membrane (PM) contact sites with the endoplasmic reticulum (ER), nuclear envelope and mitochondria at the mother‐bud neck cortex of *S. cerevisiae* ; (2) regulation of calcium ion influx at neuronal PM‐ER contact sites through modulation of interactions between the calcium release‐activated calcium channel protein 1 (Orai1) and the stromal interaction molecule 1 (STIM1) at steady state and during store‐operated calcium entry (SOCE); (3) diffusion barriers and ER‐PM crosstalk at the base‐neck cortex of post‐synaptic dendritic spines; (4) dual roles at MCS between synaptic vesicles and the pre‐synaptic PM, where septins can act both as barriers and tethers restricting and promoting synaptic vesicle release, respectively; and (5) mediation of membrane contacts between intracellular bacterial pathogens and organelles—including mitochondria and lysosomes—to promote autophagic clearance.

**Abbreviations**
AMPA: α‐amino‐3‐hydroxy‐5‐methyl‐4‐isoxazolepropionic acid (receptor)
ARP2/3: Actin‐related protein 2/3
Atg8: Autophagy‐related protein 8
Atg9: Autophagy‐related protein 9
Ca^2+^
: Calcium ion
cecER: Central cisternal ER
DG: Dentate gyrus
ER: Endoplasmic reticulum
GABARAPL2: GABA type A receptor‐associated protein like 2
GAP: GTPase‐activating protein
Gb3: Globotriaosylceramide
GDP: Guanosine diphosphate
GEF: Guanine nucleotide exchange factor
GTP: Guanosine triphosphate
GTPase: Guanosine triphosphatase
IMM: Inner mitochondrial membrane
LC3B: Microtubule‐associated protein 1A/1B‐light chain 3B
LD: Lipid droplet
MCS: Membrane contact site
Myo 5: Myosin 5
NE: Nuclear envelope
NPC: Neuronal precursor cell
NSF: N‐ethylmaleimide‐sensitive factor
N‐WASP: Neural Wiskott–Aldrich syndrome protein
OMM: Outer mitochondrial membrane
Orai1: Calcium release‐activated calcium channel protein 1
PINK1: PTEN‐induced kinase 1
PIP2: Phosphatidylinositol 4,5‐bisphosphate
PM: Plasma membrane
pmaER: PM‐associated ER
SNAP‐25: Synaptosomal‐associated protein 25
SNARE: Soluble N‐ethylmaleimide‐sensitive factor attachment protein receptor
SOCE: Store‐operated calcium entry
STIM1: Stromal interaction molecule 1
TNF‐α: Tumor necrosis factor alpha
tubER: Tubular ER
VAMP‐2: Vesicle‐associated membrane protein 2
VAP: VAMP‐associated protein
WAVE: WASP‐family verprolin homologous protein
α‐SNAP: Alpha‐soluble NSF attachment protein

## 1 Introduction

Neuronal homeostasis and physiology depend on communication and signal transduction across various cellular compartments and scales. In neurons, intracellular communication encounters unique spatial challenges, as many signals are generated locally and yet propagate globally through cellular regions that are geometrically complex and highly compartmentalized (Higley and Sabatini 2008; Terenzio et al. 2017; Kirchner and Gjorgjieva 2021). Despite these architectural constraints, ion exchange and buffering, lipid homeostasis, and organelle positioning are maintained across long distances and specialized subdomains such as axons, dendrites, presynaptic terminals, and dendritic spines (Bentley and Banker 2016; Donato et al. 2019; Guedes‐Dias and Holzbaur 2019).

How do neurons achieve intracellular communication and coordination with such spatiotemporal accuracy and efficacy? Historically, intracellular communication was thought to occur primarily through diffusion and mechanisms of active transport in the cytoplasm (Voeltz et al. 2024). More recently, however, the discovery of organelle‐organelle contacts has fundamentally reshaped our understanding of intracellular signaling, metabolism, and organelle biogenesis (Prinz et al. 2020; Voeltz et al. 2024). These processes are increasingly viewed through the perspective of a membranous continuum—a network of organelles adjoined at membrane contact sites (MCS) (Prinz et al. 2020). This interconnected web of membranes may represent a key organizational principle—a hidden advantage—that enables highly coordinated and efficient signaling across the elongated and complex architecture of neurons.

MCS are specialized regions where two organelles are closely apposed, typically within 10 to 30 nm, without undergoing membrane fusion (Scorrano et al. 2019; Prinz et al. 2020; Voeltz et al. 2024). These membrane junctions are selectively established and maintained by proteins and protein complexes collectively termed membrane tethers, which facilitate and/or mediate the exchange of ions, lipids, and other metabolites, and play critical roles in organelle dynamics and biogenesis (Prinz et al. 2020; Voeltz et al. 2024). In neurons, the endoplasmic reticulum (ER)—the most extensive intracellular organelle—forms MCS with the plasma membrane (PM), mitochondria, endolysosomes, and lipid droplets (Wu et al. 2017; Ralhan et al. 2021; Benedetti et al. 2025). Neuronal ER‐PM contacts are critically important in maintaining calcium (Ca^2+^) homeostasis and orchestrating both local and long‐range Ca^2+^ signaling events, which regulate the Ca^2+^‐dependent machinery underlying neurotransmission (Clapham 2007; Higley and Sabatini 2008; Courjaret et al. 2024). Importantly, however, MCS are not restricted to the ER. They can take place between any organelle and surrounding membranes, opening a wide range of functional possibilities that have only recently begun to be explored.

Here, we review how filaments of the septin cytoskeleton function at MCS in neurons and beyond. Septins are a fundamental component of the neuronal cytoskeleton with critical roles in neuronal morphogenesis (Ageta‐Ishihara and Kinoshita 2021; Radler and Spiliotis 2022; Alkhanjari et al. 2025). Septins are essential for the development and maintenance of the polarized and branched shapes of neurons and play important roles in the generation of neuronal compartments and structures including the axon initial segment, myelin sheaths, dendritic spines, and synapses (Ageta‐Ishihara and Kinoshita 2021; Radler and Spiliotis 2022; Alkhanjari et al. 2025). In contrast to these well‐studied structures, neuronal MCS have received less attention, and the role of septins at neuronal MCS in particular remains largely unexplored.

## 2 Septins: Cytoskeletal Filaments With Membrane‐Binding Properties

Septins are a family of GTP‐binding proteins that assemble into filamentous polymers, collectively constituting a distinct cytoskeletal network alongside microtubules, actin microfilaments, and intermediate filaments (Mostowy and Cossart 2012; Cavini et al. 2021). Septins are conserved from fungi to animals, but they are absent in terrestrial plants (Shuman and Momany 2021). Since their discovery at the cortical membrane of the mother‐bud neck of the yeast *Saccharomyces cerevisiae* (Byers and Goetsch 1976), a defining feature of the septin cytoskeleton has been its pronounced site specificity (Caudron and Barral 2009; Spiliotis 2018). Septin filaments associate with distinct subdomains of the plasma membrane, and subsets of microtubules and actin filaments, which in mammalian cells largely localize in perinuclear regions of the cytoplasm (Bridges and Gladfelter 2015; Spiliotis and Nakos 2021; Nakazawa et al. 2023). Septin filaments form higher‐order networks ranging from linear and curved bundles to rings and gauze‐like meshworks. These filamentous networks exert spatial control over the localization of membrane and cytoskeletal proteins, functioning both as selective scaffolds that facilitate protein recruitment and interactions and as barriers that impede protein exchange through steric or diffusional obstruction (Kinoshita 2006; Caudron and Barral 2009; Oh and Bi 2011; Benoit et al. 2023).

The mammalian family of septins is encoded by 13 different genes, which give rise to a multitude of paralogs and isoforms categorized into four distinct groups—SEPT2, SEPT3, SEPT6, and SEPT7—based on sequence similarity (Kinoshita 2003a). With the exception of SEPT7, all other septin groups contain multiple paralogs with ubiquitous and tissue‐specific expression patterns. Septins assemble combinatorially into heteromeric, non‐polar oligomers such as SEPT2/6/7 or SEPT2/6/7/9, which interact tail‐to‐tail to form hexamers and octamers, respectively (McMurray and Thorner 2019). These palindromic oligomers serve as the protomeric units of septin filaments, and their subunit composition can vary as paralogs within the same group can substitute for one another (Kinoshita 2003a; Rosa et al. 2020). Septin complexes of distinct subunit combinations therefore arise, each with unique localizations and functions determined by the collective properties of their constituent subunits (Garcia et al. 2011; Khan et al. 2018; Cannon et al. 2023; Stjepić et al. 2024; Perry et al. 2025). Of note, septin subunits present in stoichiometric excess of their cognate partners may form protomeric complexes and filaments of alternate composition (Sellin et al. 2011; Serrão et al. 2011; Kim et al. 2012). These non‐canonical models of assembly are poorly understood and might be the result of paralog‐ or isoform‐specific properties beyond simple differences in expression levels.

**FIGURE 1:**
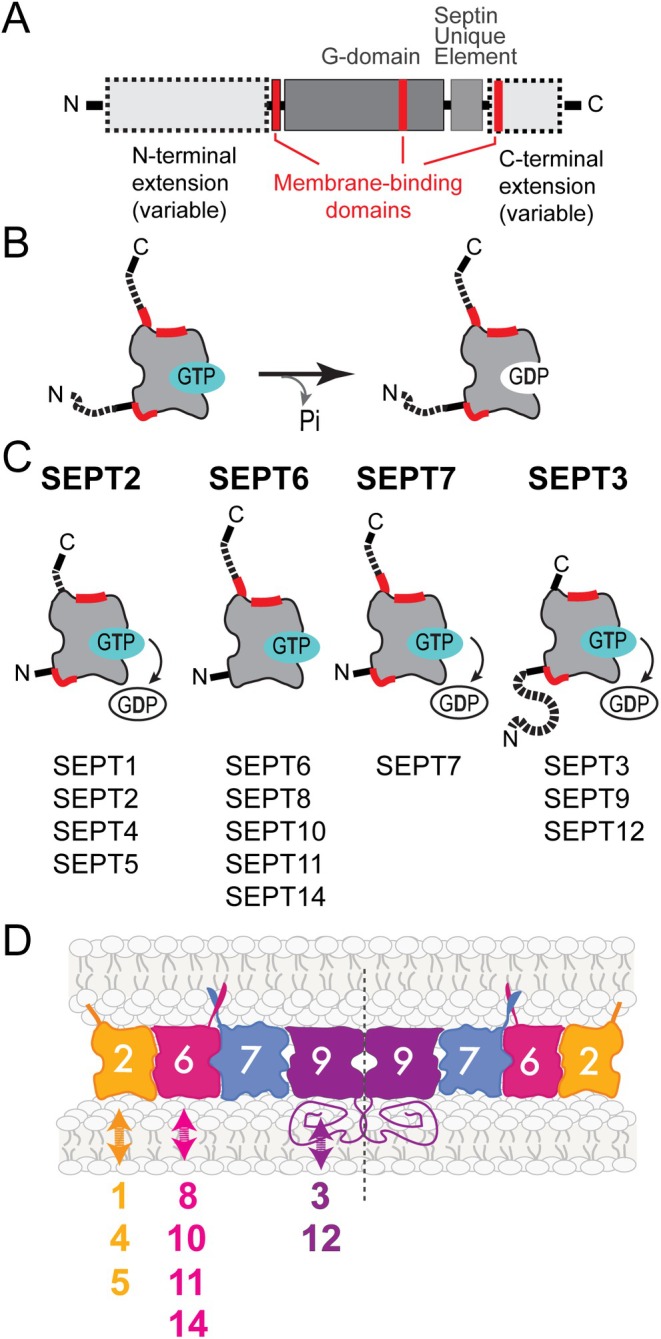
The septin family of GTP‐binding proteins. (A) Schematic of the major domains of septins. The core of each septin consists of a highly conserved GTP‐binding domain (G domain) with a P‐loop fold, flanked by N‐ and C‐terminal extensions of variable length and sequence. The G domain terminates with a C‐terminal α‐helix known as the septin unique element (SUE). Membrane‐binding regions (red) are found at three locations: Near the N‐ and C‐terminal ends of the G domain, where they consist of amphipathic helices followed by polybasic sequences, and within the C‐terminal extensions, where they form amphipathic helices or predicted transmembrane domains. (B) Structure of the septin G domain. Septins bind and hydrolyze GTP through their globular G domain. The positions of the membrane‐binding regions (red) are shown relative to the N‐ and C‐terminal ends of the G domain. (C) The human septin family comprises 13 genes, classified into four groups named after SEPT2 (SEPT1, SEPT2, SEPT4, SEPT5), SEPT6 (SEPT6, SEPT8, SEPT10, SEPT11, SEPT14), SEPT7, and SEPT3 (SEPT3, SEPT9, SEPT12). Members of the SEPT6 group are catalytically inactive—they do not hydrolyze GTP—and lack the N‐terminal G‐domain membrane‐binding region present in other groups. Instead, their C‐terminal extensions contain amphipathic helices and predicted transmembrane domains that mediate membrane association. (D) Septins assemble into non‐polar hetero‐oligomeric complexes incorporating members from each of the four groups. The prototypical unit for higher‐order polymer assembly is a hetero‐octamer consisting of two SEPT2/6/7/9 tetramers joined in a palindromic, tail‐to‐tail arrangement. Because septins within the same group are interchangeable at equivalent positions, oligomers of varying subunit composition can form, giving rise to distinct intracellular localizations and interactions. The presence of multiple membrane‐binding regions on opposite faces of the oligomer may enable septins to engage two juxtaposed membrane bilayers simultaneously, allowing single filaments, paired filaments, or bundled arrays to bridge or scaffold membrane surfaces.

Structurally, septins are related to the small GTPases of the Ras superfamily, containing a highly conserved GTP‐binding domain with variable N‐ and C‐terminal extensions, which harbor protein interaction domains such as proline‐rich sequences and alpha‐helical coiled coils (Cavini et al. 2021) (Figure 1A). Septins have variable GTPase activities, which are generally slower than that of canonical small GTPases and entirely absent in the SEPT6 group, which remains constitutively GTP‐bound (Zent and Wittinghofer 2014) (Figure 1B,C). Septin subunits assemble through their GTP‐binding domains via two alternating dimeric interfaces, termed G‐G and N‐C (Sirajuddin et al. 2007; Rosa et al. 2020; Cavini et al. 2021). The state of the guanine nucleotide (GTP vs. GDP) is critical in the early stages of septin assembly, determining the identity of the dimerizing subunits and influencing the stability of the dimeric interfaces within oligomers and polymers (Cavini et al. 2021; Brown and McMurray 2025) (Figure 1C,D). How GTP hydrolysis is regulated remains unclear, but it does not appear to involve canonical guanine nucleotide exchange factors (GEF) and GTPase‐activating proteins (GAPs).

Septins associate with membranes through three distinct types of domains: (i) polybasic motifs, which are located either at the N‐terminus or within the GTP‐binding domain and interact with phosphoinositides such as phosphatidylinositol 4,5‐bisphosphate (PIP2) and phosphatidylinositol‐4‐phosphate (Zhang et al. 1999; Song et al. 2019; Castro et al. 2020), (ii) amphipathic α‐helix motifs adjacent to the polybasic domains or within C‐terminal extensions, which mediate interactions with curved membranes (Cannon et al. 2019; Woods et al. 2021; Lobato‐Márquez et al. 2021), and (iii) transmembrane domains, which remain poorly characterized in mammalian septins (Perry et al. 2025) (Figure 1A,B). Septins associate predominately with PIP2‐containing domains of the PM and preferentially concentrate in micron‐scale domains of hyperboloid (saddle‐shaped) membrane curvature (Bridges et al. 2016; Curtis and Gladfelter 2024). Interactions with intracellular membranes have been less extensively studied, but septins have been reported to localize to the nuclear envelope (Okletey et al. 2023; Zhang et al. 2024), the Golgi complex (Song et al. 2019; Omrane et al. 2019), endolysosomes (Zander et al. 2016; Danson et al. 2018; O'Loughlin et al. 2018; Kesisova et al. 2021; Robinson et al. 2024), macropinosomes (Dolat and Spiliotis 2016), mitochondria (Mandel‐Gutfreund et al. 2011; Pagliuso et al. 2016; Sirianni et al. 2016; Shannon et al. 2025), lipid droplets (Akil et al. 2016; Moreno‐Castellanos et al. 2017; Chen et al. 2021), and autophagosomes (Barve et al. 2018). Notably, septins also assemble around bacterial pathogens, forming cage‐like structures that restrict bacterial motility and promote their degradation through autophagy (Van Ngo and Mostowy 2019).

Septins play multifaceted roles in the spatial organization of cell membranes. First, they scaffold many proteins including signaling molecules, vesicle tethering and fusion complexes, membrane remodeling proteins, and cytoskeletal regulators (Kinoshita 2006; Spiliotis and Kesisova 2021; Benoit et al. 2023). Second, they compartmentalize membranes by establishing diffusion barriers that corral proteins and partition membranes into distinct domains by selectively impeding the diffusion of proteins and lipids (Caudron and Barral 2009). Third, septins synergize with the actin and spectrin components of the membrane cytoskeleton to establish and maintain membrane domains, as has been demonstrated at the perisynaptic membrane of Bergmann glia in the cerebellum and the axon initial segment (Ageta‐Ishihara et al. 2018; Hamdan et al. 2020). Collectively, these properties position septins as versatile organizers of membrane architecture. Growing evidence indicates that septins localize to interfaces between intracellular membranes (see below). However, whether septins are *bona fide* functional components of MCS has not been systematically examined or discussed—a gap this review aims to address.

## 3 Septins at ER‐PM Contact Sites: From Yeast Diffusion Barriers to Neuronal Compartmentalization

Since their discovery in *S. cerevisiae* , budding yeast septins have provided foundational insights into septin organization and function, many of which prove relevant to neuronal morphogenesis and physiology (Barral and Mansuy 2007; Falk et al. 2019; Spiliotis and McMurray 2020). In budding yeast, septins function at a specialized subcellular interface where the PM and ER converge, establishing diffusion barriers that compartmentalize membrane proteins and maintain cellular asymmetry. Recent evidence suggests that mammalian septins may serve analogous functions in neurons, particularly at dendritic branch points and spine necks (Xie et al. 2007; Tada et al. 2007; Ewers et al. 2014).

In budding yeast, approximately 20%–60% of the PM associates closely with a specialized ER subdomain termed PM‐associated ER (pmaER), forming a 15‐to‐60 nm wide interface that lacks endocytic and exocytic events, as well as ribosomes which instead localize to the distal cytoplasmic face of the pmaER (Pichler et al. 2001; Schuck et al. 2009; West et al. 2011; Stradalova et al. 2012). The pmaER is structurally distinct from the tubular ER (tubER), central cisternal ER (cecER), and nuclear envelope (NE), though all domains are interconnected (West et al. 2011). The cecER forms contacts with both the NE and the pmaER, while the tubER spans the cytoplasm and links all ER subdomains into a continuous network (West et al. 2011). Strikingly, the pmaER is absent from the mother‐bud neck PM, while tubER extends from the mother into the bud, as the inherited ER is continuous with the mother cecER and tubER (West et al. 2011).

Septins localize to the mother‐bud neck of budding yeast, forming an hourglass‐shaped collar‐like structure that consists of filamentous bars and rungs. This architecture supports the establishment and maintenance of diffusion barriers that restrict lateral movement of both PM and ER membrane proteins from mother to daughter cell (Barral et al. 2000; Luedeke et al. 2005). Septins appear to selectively exclude the pmaER domain from entering the bud, while permitting tubER entry (Luedeke et al. 2005; West et al. 2011); note that the bud neck ER was originally characterized as non‐tubular sheet ER (Luedeke et al. 2005). Exclusion of the pmaER involves competition between septins and ER‐PM tethers for binding to the bud neck PM, which occurs in a mutually exclusive manner (Sugiyama and Kono 2024). Additionally, ER‐PM tethers are cleared from nascent bud sites prior to septin assembly through exocytic vesicle delivery (Sugiyama and Kono 2024). Polarized delivery of new membrane is thought to generate a membrane flow that moves ER‐PM tethers into the mother PM, and thereby enables septin assembly (Sugiyama and Kono 2024).

In the absence of pmaER‐PM contacts at the mother‐bud neck, septins link the PM and the tubER through indirect and direct mechanisms. Septins interact directly with the ER through binding of the C‐terminal extension of the septin subunit Shs1 to the ER resident protein Scs2, a member of the VAP protein family (Chao et al. 2014). In addition, septins organize a signaling module at the bud neck PM involving the small GTPases Bud1 and Cdc42, which are activated by the guanine exchange factors (GEFs) Bud5 and Cdc24, respectively (Clay et al. 2014). This signaling cascade recruits the formin Bud6, an actin nucleation‐promoting factor that associates with the ER (Jin and Amberg 2000; Luedeke et al. 2005; Graziano et al. 2011).

**FIGURE 2:**
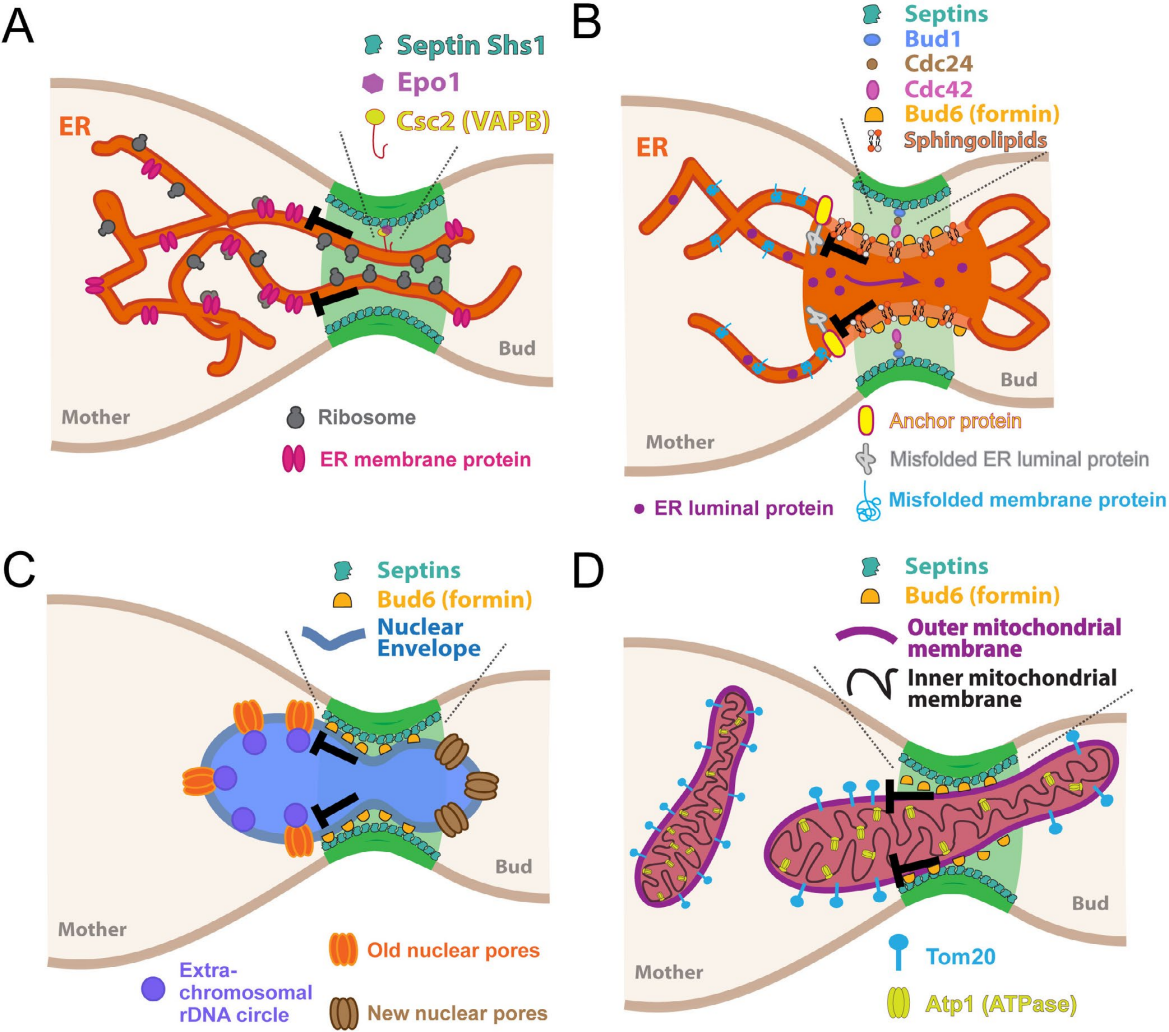
Septin functions at PM contact sites with organelle membranes in budding yeast. (A) At the mother‐bud neck cortex, the PM‐associated septin Shs1 interacts with the ER protein Csc2—A yeast homolog of the mammalian VAMP‐associated protein B (VAPB)—and the adaptor protein Epo1. At the PM–ER interface, ribosomes are spatially excluded, and PM‐associated septins crosstalk with the ER to restrict the free diffusion of transmembrane proteins such as the translocon channel, which co‐translationally threads nascent polypeptides into and across the ER membrane. (B) The lateral diffusion of misfolded ER transmembrane proteins—as well as misfolded lumenal proteins anchored to an ER membrane protein—is restricted at the mother‐bud neck, resulting in their asymmetric retention in the mother cell. By contrast, lumenal ER proteins diffuse freely between the mother‐ and bud‐localized ER domains, although overall diffusion rates might be decreased due to ER partitioning. The ER membrane diffusion barrier depends on the formation of a sphingolipid‐enriched domain in the cytoplasmic leaflet of the ER membrane, juxtaposed to the mother‐bud neck cortex. PM‐associated septins scaffold a signaling module involving the sequential recruitment and activation of the small GTPases Bud1 and Cdc42 by their respective guanine nucleotide exchange factors (GEFs) Bud5 and Cdc24. This cascade culminates in the recruitment of the formin Bud6, which is proposed to stabilize the sphingolipid‐enriched ER domain by locally promoting Actin nucleation. (C) Cortical septins are also required for restricting the lateral diffusion of nuclear pore complexes and nuclear pore‐associated extrachromosomal ribosomal DNA (rDNA) circles within the domain of the nuclear envelope confined to the mother compartment. This function depends on the recruitment of the formin Bud6. (D) Through a related mechanism, septins promote the formation of a diffusion barrier in the inner mitochondrial membrane, limiting the lateral movement of the inner membrane‐anchored ATP synthase subunit Atp1 across the mother‐bud neck cortex. A similar diffusion barrier exists in the outer mitochondrial membrane under conditions of stress, but it is unclear whether it is septin‐dependent.

Septin association with the ER is critical for maintaining a barrier in the ER membrane that restricts the diffusion of integral ER membrane proteins such as the translocon channel from entering the bud (Luedeke et al. 2005; Clay et al. 2014) (Figure 2A,B). Lumenal proteins are largely able to diffuse freely (Luedeke et al. 2005), but the rate of diffusion appears to increase in yeast strains with a septin temperature‐sensitive mutant, which might be indicative of a septin function in partitioning the pmaER (Sugiyama and Tanaka 2019). Although the molecular mechanism by which septins physically impede protein diffusion on the cytoplasmic leaflet of the ER membrane remains unclear, the ER diffusion barrier depends on sphingolipid biosynthesis and the formation of a sphingolipid‐rich domain in the bud neck ER (Clay et al. 2014). Septins are thought to stabilize this ER membrane domain—potentially formed through phase separation of sphingolipids into a liquid ordered domain—by recruiting the formin Bud6 and thereby promoting actin assembly on the ER (Clay et al. 2014) (Figure 2B). Notably, this septin‐dependent ER barrier is also a region of increased membrane thickness, which arises from the accumulation of ceramides—sphingolipid precursors with long and saturated fatty acid chains—that selectively hinder the passage of proteins with short transmembrane domains (Prasad et al. 2020).

The septin‐dependent ER membrane diffusion barrier prevents misfolded proteins from entering the daughter cell, thereby playing a critical role in asymmetric aging under conditions of ER stress (Clay et al. 2014). Though the membrane diffusion barrier primarily interferes with membrane proteins, both transmembrane and lumenal misfolded proteins are prevented from passing through the bud neck (Clay et al. 2014) (Figure 2B). This discrepancy suggests there could be a yet unknown anchor attaching them to the membrane (Clay et al. 2014).

This function in asymmetric aging extends to nuclear envelope compartmentalization during mitosis (Figure 2C). At the onset of anaphase, when the nucleus enters into the bud, the nuclear envelope becomes similarly partitioned through a septin‐ and Bud6‐dependent diffusion barrier that restricts lateral mobility of nuclear pores (Shcheprova et al. 2008). Nuclear pores from the mother cell are excluded from the bud portion of the nuclear envelope, establishing nuclear pore asymmetry (Shcheprova et al. 2008). This asymmetry contributes to aging, as nuclear pores are bound to extrachromosomal ribosomal DNA circles, which accumulate in the mother cell over successive divisions (Falcón and Aris 2003; Shcheprova et al. 2008).

Similar to the nuclear membrane and the ER, a mitochondrial diffusion barrier is present at the bud neck, restricting the movement of outer and inner membrane proteins while allowing matrix proteins to diffuse freely (Yoshii and Barral 2024) (Figure 2D). The inner mitochondrial membrane (IMM) has a constitutively present diffusion barrier, which is partially dependent on both septins and Bud6 (Yoshii and Barral 2024). In contrast, the outer mitochondrial membrane (OMM) has a diffusion barrier only under conditions of cell stress, and it is unclear whether it is septin‐dependent (Yoshii and Barral 2024).

**FIGURE 3:**
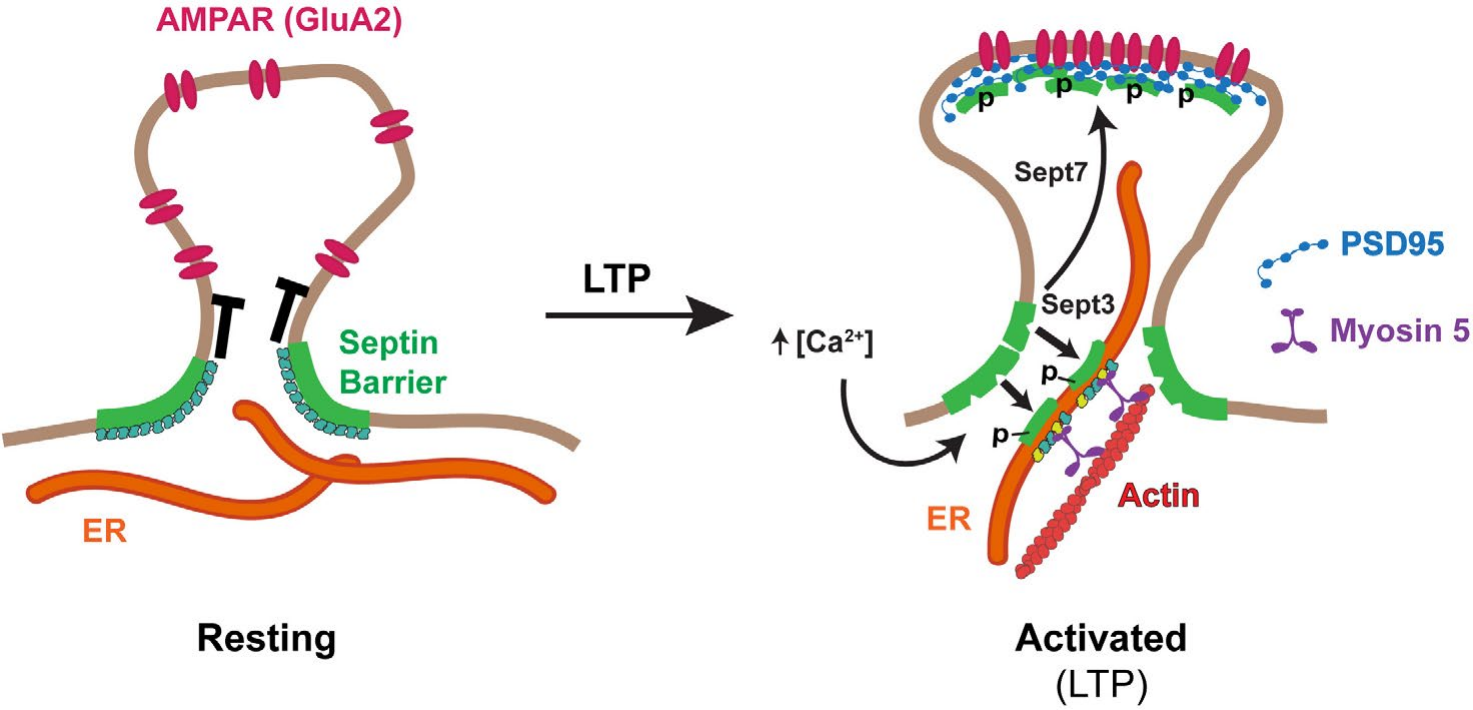
Septin functions at the PM–ER interface of dendritic spines. Septins are enriched at PM domains of curvature that demarcate the base‐neck of dendritic spines, where they restrict the free lateral diffusion of AMPA‐type glutamate receptors. In Sept7‐depleted neurons, the diffusional mobility of glutamate receptors into the dendritic shaft membrane is enhanced, indicating that Sept7‐containing complexes limit the lateral diffusion of post‐synaptic membrane proteins. Under conditions of long‐term potentiation (LTP) induced by sustained synaptic stimulation— which elevates post‐synaptic Ca^2+^ concentrations— phosphorylation of Sept7 and Sept3 triggers their redistribution to distinct spine membrane compartments. Phosphorylated Sept7 localizes to dendritic spine heads, where it associates with PSD‐95 and promotes its synaptic stability by restricting its lateral mobility. Phosphorylated Sept3 relocalizes to the ER membrane, where it recruits the myosin 5 motor (MYO5A), which in turn facilitates ER membrane translocation along actin filaments into the spine.

The principles of septin‐mediated compartmentalization discovered in yeast may extend to mammalian neurons, where ER diffusion barriers have been reported at dendritic branch points (Wang et al. 2012)—sites known to be enriched in septins (Xie et al. 2007; Tada et al. 2007; Li et al. 2009; Cho et al. 2011; Kaplan et al. 2017). ER cargo proteins exhibit limited mobility at branch points, which enhances local delivery of nascent proteins to the PM (Wang et al. 2012). At dendritic spine necks, a septin‐dependent diffusion barrier appears to restrain the intramembrane flow of AMPA‐type glutamate receptors as well as transmembrane and inner membrane leaflet proteins (Ewers et al. 2014) (Figure 3). Although it remains unknown whether mammalian septins associate with neuronal ER to constrain ER membrane protein diffusion, as has been observed in yeast, recent studies indicate that septins can directly hinder the diffusional mobility of PM proteins and phospholipids (Pacheco et al. 2022; Chauvin et al. 2025).

Interestingly, new work shows that septins associate with neuronal ER. Sept3 localizes to both spine necks and smooth ER in the hippocampal dentate gyrus (DG) in mice (Ageta‐Ishihara, Fukazawa, et al. 2025; Ageta‐Ishihara, Mizukami, et al. 2025). Following electroconvulsive stimulation, Sept3 becomes phosphorylated and increasingly enriched on smooth ER, where it associates with the myosin motor MYO5A and promotes ER entry into dendritic spines (Ageta‐Ishihara, Mizukami, et al. 2025) (Figure 3). Similarly, late‐phase long‐term potentiation increases Sept3 expression in mouse hippocampal DG granule cells, where it facilitates MYO5‐dependent smooth ER movement into spines—a process critical for memory consolidation (Ageta‐Ishihara, Fukazawa, et al. 2025). These findings suggest that Sept3 coordinates PM and ER membrane dynamics at the base of dendritic spines, though direct evidence for PM‐bound septin interactions with neuronal ER awaits further investigation.

In addition to ER‐PM junctions, septin‐dependent principles of membrane compartmentalization may extend to other ER‐derived membrane domains. Lipid droplets (LDs) originate from the ER and maintain connection through MCS during their maturation (Grippa et al. 2015). These ER‐LD contact sites include a diffusion barrier that stabilizes the contact and prevents unrestricted exchange of ER and LD components (Grippa et al. 2015). Consistent with this architectural role, septins regulate LD biogenesis and dynamics in non‐neuronal cells (Akil et al. 2016; Chen et al. 2021; Song et al. 2022). The ER‐resident protein FIT2 binds SEPT7 via cytosolic loops, and this interaction is required for nascent LD formation and droplet growth (Chen et al. 2021). SEPT9 has also been implicated in LD enlargement and positioning, as well as in regulating LD–lysosome association, coordinating organelle crosstalk and lipid metabolism (Akil et al. 2016; Song et al. 2022).

Overall, yeast septins function as specialized ER‐PM adaptors that organize the ER into distinct membrane compartments at spatially defined PM sites by enabling the formation of diffusion barriers. While it remains unclear whether neuronal septins function analogously, growing evidence points to conserved principles of septin‐mediated membrane compartmentalization across evolution (Caudron and Barral 2009; Spiliotis and McMurray 2020). Importantly, septins differ functionally from canonical ER‐PM tethers, which primarily mediate lipid and calcium exchange without impacting lateral diffusion within membranes. By forming diffusion barriers at MCS, septins could help maintain the position of tethers or lipid microdomains, indirectly supporting lipid exchange between organelles and extending principles observed at ER‐PM junctions to other MCS.

## 4 Septins Regulate Store‐Operated Calcium Entry (SOCE) at ER‐PM Contact Sites

Neuronal store‐operated calcium entry (SOCE), which replenishes ER calcium levels, generates local and long‐range Ca^2+^ signals that regulate gene transcription, synaptic plasticity, and axonal growth (Majewski and Kuznicki 2015). The stromal interaction molecule 1 (STIM1) is an ER resident protein that localizes to ER regions adjacent to the PM upon sensing low ER calcium levels (Liou et al. 2005; Zhang et al. 2005; Wu et al. 2006). STIM1 binds directly to the C‐terminus of Orai1, the PM‐associated pore forming subunit of the calcium release‐activated calcium channel, triggering its opening and calcium influx (Park et al. 2009; Zhou et al. 2016). ER stores are then refilled via sarco/endoplasmic reticulum calcium ATPase (SERCA) pumps, which results in dissociation of STIM1 from Orai1 (Luik et al. 2006; Wu et al. 2006; Prakriya and Lewis 2015). Efficient SOCE requires the rapid and tightly regulated assembly of STIM1‐Orai1 clusters at ER‐PM contact sites, a process facilitated by lipid microdomains of the PM enriched in phosphatidylinositol 4,5‐bisphosphate (PIP2) (Walsh et al. 2010; Calloway et al. 2011; Maléth et al. 2014). Under resting conditions, Orai1 channels are inhibited at these PM domains to prevent constitutive calcium influx, as elevated intracellular calcium leads to cellular dysfunction (Shim et al. 2015; Bakowski et al. 2021). Thus, SOCE regulation depends on a balance between mechanisms that promote STIM1‐Orai1 assembly upon ER calcium depletion and mechanisms that inhibit channel activation in the absence of store depletion.

**FIGURE 4:**
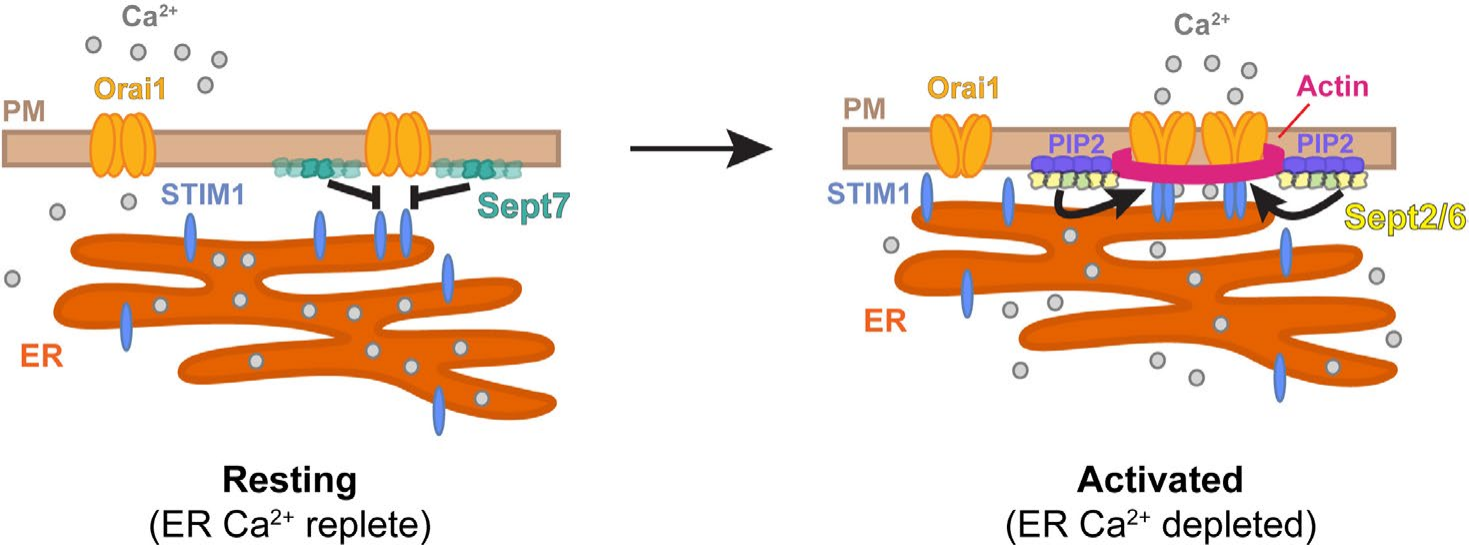
Septin regulation of Ca^2+^ influx at PM‐ER contact sites. In the resting state (left) and under conditions of sufficient ER Ca^2+^ levels, Sept7 plays a key role in preventing Ca^2+^ influx by limiting STIM1‐mediated activation of Orai1 channels. When ER Ca^2+^ stores are depleted—for example, following neuronal activation—Septins from the Sept2 and Sept6 groups assemble at PIP2‐enriched domains of the PM, which demarcate the formation of an actin ring that corrals clusters of Orai1. At ER‐PM contact sites, septins regulate the spatial organization of PIP2 and Orai1, promoting STIM1‐Orai1 interactions and facilitating Ca^2+^ influx.

Septins both facilitate and, intriguingly, inhibit STIM1‐Orai1 mediated SOCE at ER‐PM sites (Deb and Hasan 2016) (Figure 4). Septins of the SEPT2 group have emerged as enhancers of SOCE and STIM1‐Orai1 clustering in membrane microdomains in multiple organisms and non‐neuronal cell types (Sharma et al. 2013; Katz et al. 2019; de Souza et al. 2021; Tripoli and Smyth 2024). Rather than interacting directly with STIM1 or Orai1 (Katz et al. 2019), SEPT2 group septins enable a re‐organization of the local lipid and cytoskeletal environment to facilitate STIM1‐Orai1 interaction (Sharma et al. 2013; de Souza et al. 2021). In mammalian cells, SEPT4—a SEPT2 group septin—localizes to the PM following calcium depletion and is required for efficient Orai1 clustering (Sharma et al. 2013). However, SEPT4 and Orai1/STIM1 do not colocalize at the PM (Sharma et al. 2013). Instead, SEPT4 concentrates in PM domains following calcium depletion, which coincide temporally with STIM1 redistribution, and are spatially adjacent to ER‐PM junctions (Sharma et al. 2013). Depletion of SEPT2 group members SEPT4 and SEPT5, and to a lesser extent SEPT2, impairs STIM1‐Orai1 clustering (Sharma et al. 2013; de Souza et al. 2021), but no direct binding of septins with STIM1‐Orai1 proteins has been observed (Katz et al. 2019). In *Drosophila* neurons, depletion of SEPT2 group septins dSept4 and dSept1, as well as that of the SEPT6 group septin dSept2, leads to attenuated SOCE, indicating a similar role to the mammalian SEPT2 group septins in promoting SOCE, (Deb and Hasan 2019); note that with the exception of Pnut (dSept7), *Drosophila* septin paralogs are classified differently than their mammalian counterparts under the SEPT2 (dSept1, dSept4) and SEPT6 (dSept2, dSept5) groups (Kinoshita 2003b).

The mammalian SEPT2 group septins SEPT4 and SEPT5 promote STIM1‐Orai1 interactions at ER‐PM junctions by regulating the local organization of the actin cytoskeleton and PIP2, which is critical for STIM1 clustering and proper Orai1 activation; PIP2 depletion drastically reduces SOCE response (de Souza et al. 2021). Following store depletion, SEPT4 and SEPT5 accumulate with PIP2 into domains which surround Orai1 clusters (Sharma et al. 2013) (Figure 4). PIP2 promotes CDC42 recruitment, which in turn enables activation of actin‐nucleation regulators N‐WASP/WAVE and ARP2/3 (de Souza et al. 2021). This triggers actin remodeling into transient ring‐like structures surrounding STIM1 clusters at ER‐PM junctions. Disruption of SEPT4, CDC42, or ARP2/3 impairs STIM1 clustering, Orai1 recruitment, and SOCE, demonstrating that septin‐mediated actin remodeling is essential for optimal STIM1‐Orai1 organization (de Souza et al. 2021). As septin filaments containing SEPT2 restrict diffusion of PIP2 in the PM inner leaflet (Pacheco et al. 2022), and can remodel lipid bilayers into ring‐like and tubular structures (Tanaka‐Takiguchi et al. 2009; Vial et al. 2021), it is unclear whether septins play a role in actin organization directly and/or through PIP2 re‐organization. Moreover, it is also unknown whether PIP2 reorganization occurs upstream of septin function, as PIP2 promotes septin assembly on membranes (Bertin et al. 2010; Badrane et al. 2012).

In contrast to septins of the SEPT2 group, SEPT7 acts as a negative regulator of SOCE in neurons. Work in *Drosophila* has shown that overexpressing dSept7 (Pnut) decreases SOCE calcium influx (Deb et al. 2016). Conversely, reducing dSept7 levels can rescue impaired SOCE (Deb et al. 2016). Heterozygous deficiency of dSept7 can compensate for loss of dSTIM proteins, partially restoring calcium influx (Deb et al. 2016). Importantly, the decrease of dSept7 also increases basal influx of calcium, independently of its function in SOCE regulation. This calcium influx occurs through Ca^2+^ store‐independent activation of dOrai channels, indicating that dSept7 inhibits dOrai constitutively (Deb et al. 2016). In human neuronal precursor cells (NPCs), SEPT7 knockdown did not affect SOCE but increased basal calcium influx in NPCs, which, unlike in *Drosophila*, was dependent on STIM1 (Deb et al. 2020). This basal influx appears to occur due to reorganization of STIM1 and Orai1, and an aberrant increase of STIM1 at the cell surface under resting conditions (Deb et al. 2020). Knockdown of Orai1, however, did not fully rescue the increase in calcium influx caused by SEPT7 knockdown, indicating that SEPT7 may affect calcium channels beyond Orai1 (Deb et al. 2020). Though it has not been established whether SEPT7 interacts with Orai1 channels, SEPT7 interacts with membranes via its N‐terminal polybasic region, and without this domain, SEPT7 does not inhibit Orai1 (Deb et al. 2020).

In mouse Purkinje neurons, in vivo knockout of Sept7 rescues innervation, gene expression, and motor coordination deficits caused by STIM1 knockout (Dhanya and Hasan 2021). In mouse adipocytes, Sept7 has also been shown to restrain SOCE and influence adipogenesis and lipolysis, suggesting that Sept7's role in regulating SOCE‐linked signaling extends to metabolic processes in non‐neuronal cell types (Xu et al. 2025). Consistent with the inhibitory function of Sept7 in SOCE, recent work showed that calcium signaling is upregulated in a septin‐dependent manner in mouse models of Alzheimer's disease (Princen et al. 2024). In these mice, septin filament assembly on the neuronal PM is disrupted, leading to unregulated calcium signaling (Princen et al. 2024). Remarkably, a pharmacological compound (RM127) that restores septin assembly by stabilizing the heterodimeric Sept6‐Sept7 interface rescued calcium homeostasis and improved synaptic plasticity, ameliorating pathological symptoms (Princen et al. 2024). In a phase 2 clinical trial, RM127 improved patient memory, but the trial was discontinued due to off‐target liver toxicity (Nuytten et al. 2025).

SEPT7 is a ubiquitous and requisite subunit of all septin complexes as the sole member of the SEPT7 group, raising the question of how SEPT7 inhibits SOCE at ER‐PM contacts while SEPT2 and SEPT6 group septins have the opposite function. A potential explanation is that SEPT7 is in heteromeric complexes with members of SEPT2 and SEPT6 groups other than the paralogs whose expression was targeted. In *Drosophila* neurons, loss of SEPT6 group septins attenuates SOCE in a manner similar to SEPT2 group depletion, suggesting that SEPT2 group septins promote SOCE as heteromeric complexes (Deb and Hasan 2019). However, upregulation of constitutive calcium influx in dSept7 depleted neurons requires the SEPT2 group septins dSept1 and dSept4, indicating that *Drosophila* septin heterodimers function in the absence of dSept7 (Pnut) in regulating calcium influx (Deb and Hasan 2019). This is difficult to reconcile with evidence from other work showing that the five *Drosophila* septins assemble into two distinct complexes, Sept1/2/Pnut and Sept4/5/Pnut, both of which contain the SEPT7 homolog Pnut (Stjepić et al. 2024). In mammalian cells, SEPT2 group septins and SEPT7 may have distinct functions depending on the subunit composition of their respective heteromeric complexes. Alternatively, SEPT2 group septins may form non‐canonical complexes that are tissue‐specific and do not contain SEPT7. In support of this possibility, the mammalian SEPT1 has been previously reported to localize and function independently of SEPT7 in the Golgi complex of mammalian cells (Song et al. 2019).

## 5 Septin Regulation of Presynaptic Membrane Contacts

Neuronal transmission relies on the exocytosis of synaptic vesicles at presynaptic active zones, specialized regions of the PM containing scaffolding proteins and filamentous structures that organize vesicles and link them to the membrane and to each other (Südhof 2012). Although it remains unknown whether septins are components of the presynaptic filamentous network, a large body of evidence indicates that they play key roles in organizing membrane contacts of presynaptic zones and in regulating the calcium‐dependent docking, priming, and fusion of synaptic vesicles (Ageta‐Ishihara and Kinoshita 2021; Radler and Spiliotis 2022; Alkhanjari et al. 2025).

In the rat auditory brain stem, septins are critical for the development and maturation of the calyx of Held synapse, a large, fast‐acting excitatory synapse (Yang et al. 2010). During early calyx development, Sept5 colocalizes with presynaptic active zone proteins and impedes synaptic vesicles from docking tightly, acting as a spatial barrier between synaptic vesicles and active zone docking sites (Yang et al. 2010). Consistent with an inhibitory function, the septin‐stabilizing compound forchlorfenuron inhibits both spontaneous and evoked neurotransmission (Tokhtaeva et al. 2015) and reduces the rate of synaptic vesicle recycling in mouse motor neurons (Grigoryev et al. 2019). In mouse hippocampal neurons, however, loss of Sept3 does not affect synaptic vesicle recycling, indicating that some septin paralogs may be dispensable or functionally redundant in certain contexts (Tsang et al. 2008). In mature rat neurons, Sept8 localizes to the presynaptic zone, where it associates with synaptic vesicles, and primes them for fusion with the PM (Ito et al. 2009).

Mechanistically, synaptic exocytosis is initiated by vesicle docking through membrane tethering complexes and culminates in membrane fusion driven by the soluble N‐ethylmaleimide‐sensitive factor (NSF) attachment protein receptor (SNARE) proteins (Ungermann and Kümmel 2019; Jahn et al. 2024). Vesicular R‐SNAREs engage Q‐SNAREs on the PM, lowering the energy barrier for fusion by opposing the two membranes and inducing a hemifusion intermediate (Martens and McMahon 2008; Jahn et al. 2024). Membrane tethering complexes and the SNARE machinery together ensure the specificity and efficiency of vesicle fusion and are tightly regulated by factors that inhibit, prime, and recycle SNAREs. Septins have emerged as key regulators of all phases of this process through their interactions with both membrane tethers and SNAREs.

Septins were isolated from rat brain extracts as binding partners of the exocyst, a hetero‐octameric membrane tethering complex that controls exocytosis (Hsu et al. 1998; Mei and Guo 2018). In differentiating PC12 neuroendocrine cells, Sept2—formerly known as Nedd5—associates and acts synergistically with the exocyst complex in neurite outgrowth (Vega and Hsu 2003). Subsequently, studies across organisms have established that septins spatially control exocyst assembly. In budding and fission yeasts, septins are required for proper exocyst positioning with respect to the plane of cytokinesis and the incipient cell wall—the septum, from which septins derive their name (Dobbelaere and Barral 2004; Martín‐Cuadrado et al. 2005; Pérez et al. 2015; Singh et al. 2025). In *Magnaporthe oryzae*, the fungal pathogen that causes rice blast disease, the exocyst complex assembles in a septin‐dependent manner at the base of the appressorium, which is a protrusive structure that punctures the plant cuticle (Gupta et al. 2015). In mammalian cells, septins similarly ensure correct exocyst localization to the midbody during abscission, the terminal step of cell division that requires supply of new membrane and proteins (Estey et al. 2010, 2013). Despite these well‐established roles in exocyst positioning, the extent to which septins directly participate in membrane docking and tethering during the early stages of exocytosis remains poorly understood.

Septin functions in SNARE‐mediated membrane fusion are better characterized mechanistically, and septins have been shown to both promote and inhibit vesicle fusion depending on septin paralog and physiological context. As positive regulators, septins stabilize Q‐SNARE complexes at the PM and facilitate engagement with vesicular R‐SNAREs. Partial depletion of SEPT2 and SEPT7, or of SEPT2 alone, decreases the rate of protein secretion in HEK293 cells (Tokhtaeva et al. 2015). Forchlorfenuron disrupts SNARE complex assembly in PC12 cells, reducing SNAP‐25 levels and dissociating syntaxin‐1 from its SNARE partners (Tokhtaeva et al. 2015). Sept2 also interacts with Munc18 and NSF, which respectively promote SNARE complex assembly and disassembly, implicating Sept2 in SNARE complex turnover (Tokhtaeva et al. 2015). Sept8 binds the Qa‐SNARE syntaxin1A, anchoring it at the presynaptic PM (Ito et al. 2009). On synaptic vesicles, Sept8 associates with vesicle‐associated membrane protein 2 (VAMP‐2), displacing synaptophysin and thereby freeing VAMP‐2 to engage syntaxin1A for fusion (Edelmann et al. 1995; Pennuto et al. 2003; Ito et al. 2009).

In contrast to Sept2 and Sept8, Sept5 acts as a negative regulator of exocytosis. Sept5 associates with both presynaptic membranes and synaptic vesicles, suppressing exocytosis by acting as a physical barrier to SNARE complex formation (Beites et al. 1999). Expression of a dominant‐negative Sept5 mutant in HIT‐T15 cells enhances exocytosis, consistent with an inhibitory role (Beites et al. 1999). Molecularly, Sept5 binds directly to syntaxin‐1A at the α‐SNAP‐binding region, competing with α‐SNAP for occupancy (Beites et al. 1999). Notably, VAMP‐2 and SNAP‐25 can still associate with syntaxin‐1A in the presence of Sept5, suggesting that Sept5 modulates rather than abolishes SNARE assembly (Beites et al. 2005). This inhibitory activity is subject to regulation. Phosphorylation of Sept5 by the cyclin‐dependent kinase 5 decreases binding to syntaxin‐1, providing a signaling‐dependent mechanism for tuning exocytosis (Taniguchi et al. 2007; Werner and Yadav 2023). Collectively, these findings indicate that septins are dynamic modulators of SNARE function that coordinate vesicle fusion spatiotemporally. The opposing activities of Sept2 and Sept5 at presynaptic membranes parallel the antagonistic roles of Sept2 and Sept7 in SOCE, and are consistent with their incorporation into distinct neuronal complexes—Sept5/11/7 and Sept2/6/7—that likely execute divergent functions at the synapse (Xie et al. 2007; Radler et al. 2023).

## 6 Septin Functions at Host‐Pathogen Membrane Contacts

Septins contribute to cell‐autonomous immunity by participating in the interactions of infectious pathogens with host cells, and coordinating their spatial relationships with intracellular organelles (Mostowy and Shenoy 2015). By forming dynamic cage‐like structures around cytosolic bacteria and organizing membrane contacts with mitochondria, autophagosomes, and lysosomes, septins act as dynamic organizers of membrane contact sites, which in turn are critical for bacterial containment and destruction (Van Ngo and Mostowy 2019).

Autophagy—the lysosome‐dependent degradation pathway by which cells recycle intracellular material—plays a central role in defense against intracellular bacteria (Levine et al. 2011; Shibutani et al. 2015; Vargas et al. 2023). During infection, selective autophagy recognizes and sequesters specific substrates, which results in the isolation and targeting of invading bacteria for degradation. This process depends on the formation and regulation of membrane contacts, and bacteria have evolved diverse mechanisms to evade it (Huang and Brumell 2014).

Septins are integral for selective autophagy of *Shigella flexneri* , a World Health Organization priority pathogen that causes severe dysentery. Septins associate with micron‐scale curvature presented by dividing *S. flexneri* cells, binding cardiolipin—an anionic, cone‐shaped phospholipid enriched in highly curved membranes such as the cristae of the inner mitochondrial membrane (Krokowski et al. 2018; Paradies et al. 2019). Following bacterial recognition, septins assemble into cage‐like structures around the bacterial bodies, restricting movement driven by bacterial actin tails and thereby limiting cell‐to‐cell spread (Mostowy et al. 2010). Cage assembly is stimulated by tumor necrosis factor alpha (TNF‐a), a cytokine critical for cell host defense, and occurs preferentially on bacteria that are actively dividing (Krokowski et al. 2018), metabolically active (Sirianni et al. 2016; López‐Jiménez et al. 2025), and targeted to autophagy (Mostowy et al. 2010). Among septin paralogs, SEPT2, SEPT7, and SEPT9 are each required for cage formation, whereas SEPT11 is dispensable in cells expressing SEPT6, revealing a functional specialization and/or redundancy among septin complexes in the assembly into bacterial cages (Mostowy et al. 2010; Sirianni et al. 2016). Thus, septins target intracellular bacteria in a highly selective manner that depends on the biophysical and metabolic state of the bacterium.

Septin cages containing SEPT7 inhibit *Shigella* from replication and cooperate with autophagic receptors p62 and LC3B to prevent entrapped bacteria from dividing (Sirianni et al. 2016; Krokowski et al. 2018). Depletion of SEPT2 or SEPT9 markedly reduces p62 recruitment to bacteria, implicating septins in the nucleation of the autophagosome membrane at the bacterial surface (Mostowy et al. 2010; Lobato‐Márquez et al. 2023). Interestingly, in *S. cerevisiae* , septins relocalize to discrete ER‐associated PM patches upon nutrient starvation and function in early autophagosome formation by interacting with the autophagy proteins Atg8 and Atg9 (Barve et al. 2018; Perucho‐Jaimes et al. 2023), suggesting an evolutionarily conserved role for septins in phagophore initiation. Beyond scaffolding autophagosome nucleation at bacterial membranes, septins may promote fusion of the caged bacteria with lysosomes, as lysosomal and autophagic function are required for septin‐mediated clearance of cytosolic bacteria (Krokowski et al. 2018).

Septins also localize to membrane contacts between mitochondria and *Shigella*, using mitochondrial membranes as a platform for cage assembly (Sirianni et al. 2016). In uninfected cells, septins localize to mitochondrial fission sites associated with ER tubule contacts, interacting with membrane and cytoskeletal components of the membrane fission machinery (Pagliuso et al. 2016; Sirianni et al. 2016; Mageswaran et al. 2023; Shannon et al. 2025). In infected cells, electron microscopy has revealed that mitochondria are in close proximity to bacterial membranes encased by septin filaments (Sirianni et al. 2016). Functionally, mitochondrial dynamics modulate septin cage formation. Promoting mitochondrial elongation by blocking fusion increases the number of caged bacteria, while inducing fragmentation by inhibition of fusion reduces it (Sirianni et al. 2016). Given that mitochondrial elongation can enhance autophagy while protecting mitochondria from mitophagy (Rambold et al. 2011), these findings suggest that septin cages serve as organizational scaffolds that coordinate membrane contacts among bacteria, autophagosomes, lysosomes, and mitochondria to facilitate bacterial clearance. The importance of septin cages in host defense is underscored by the discovery that *Shigella* encodes multiple effectors dedicated to disrupting cage entrapment; OspC subverts septin cage assembly by promoting the ubiquitination and ADP‐riboxanation of SEPT9, disrupting SEPT9 heteromerization with SEPT6 and SEPT7, while OspG interferes with septin ubiquitination (Xian et al. 2024; Tang et al. 2026).

Septin roles at the interface of autophagosomes with mitochondria may be conserved in neuronal cells, as Sept3—a brain enriched septin—possesses Atg8‐interacting motifs. Sept3 binds to Atg8 family members GABARAPL2 (Nakahira et al. 2010; Tóth et al. 2022) and LC3B, and is present on LC3‐positive autophagic structures and mitochondria (Tóth et al. 2022). Notably, Sept3 association with LC3B increases following both autophagy and mitophagy induction (Tóth et al. 2022). Although Sept3 does not appear to associate with the early mitophagy marker PINK1 (Tóth et al. 2022), these data suggest a potential role for Sept3 in regulating macroautophagy in the brain.

In the context of host defense against pathogens, septin assembly at MCS occurs not only on internalized bacteria but also at points of pathogen entry and egress. At the PM, septins restrict the entry of *Pseudomonas aeruginosa*, a multidrug‐resistant pathogen (Aigal et al. 2022). This bacterium exploits interactions between a bacterial surface lectin and the glycosphingolipid Gb3 to initiate lipid‐zipper‐mediated internalization, as progressive membrane apposition leads to bacterial engulfment (Aigal et al. 2022). SEPT2/6/7 enhance membrane rigidity and actin assembly at these zippering sites, a restriction mechanism that is actively subverted by the *P. aeruginosa* effector ExoT, which disintegrates septin filaments in part through GAP‐mediated inactivation of host targets (Aigal et al. 2022). Similarly, SEPT11 restricts internalization of *Listeria monocytogenes* , a Gram‐positive foodborne pathogen, in non‐phagocytic cells, which is mediated by an interaction of the bacterial surface protein InlB with the host cell receptor Met (Mostowy et al. 2009). Paradoxically, other septin paralogs—including SEPT2 and SEPT9—promote *Listeria* entry as well as that of enteropathic *Escherichia coli* , pointing to paralog‐specific and context‐dependent roles in pathogen internalization (Mostowy et al. 2009; Scholz et al. 2015). In cells infected by the vaccinia virus, a member of the poxvirus family, SEPT2, SEPT7, SEPT9, and SEPT11 are recruited to the PM sites of viral egress after fusion of viral particles with the PM, forming cages around the enveloped virus (Pfanzelter et al. 2018). Similar to their role in bacterial entrapment, these cages limit viral particle release and spread to neighboring cells. Together, these findings establish septins as broad organizers of pathogen‐associated membrane contacts across diverse infection contexts, acting at multiple stages of the host‐pathogen interface.

## 7 Rethinking Septins at MCS: Principles and Mechanisms of a Membrane Tethering Code

Despite early findings in budding yeast suggesting crosstalk between PM‐bound septins at the mother‐bud neck and the ER, septins are generally not considered MCS tethers. This is likely a reflection of several compounding limitations in the field. Septins are predominately viewed as filamentous proteins that associate with the PM, actin, and microtubules, and evidence for their association with intracellular organelles and endomembranes has remained sparse. Their localization to MCS has been largely unexplored, and prevailing structural models of septin assembly assume association with a single lipid bilayer, with all available membrane‐binding domains of septin subunits engaging in cis interactions. The possibility that individual septin filaments, or bundles thereof, could make trans interactions with two opposing membranes has not been seriously considered.

Here, upon revisiting both previous and recent findings, it becomes clear that septins fit the broader definition of MCS tethers—proteins that enhance the proximity of two membrane compartments. Moreover, septins exhibit three hallmarks shared broadly by *bona fide* tethers and MCS (Prinz et al. 2020): first, they serve functions beyond simply linking two membranes, such as scaffolding or corralling integral or peripheral membrane proteins; second, septin‐associated MCS contain additional proteins that act as tethers; and third, septins associate with multiple distinct MCS. While existing evidence supports these three trends, more work is necessary to map septins in the intracellular landscape of MCS, and to determine systematically their properties, locations, and specificities.

**FIGURE 5:**
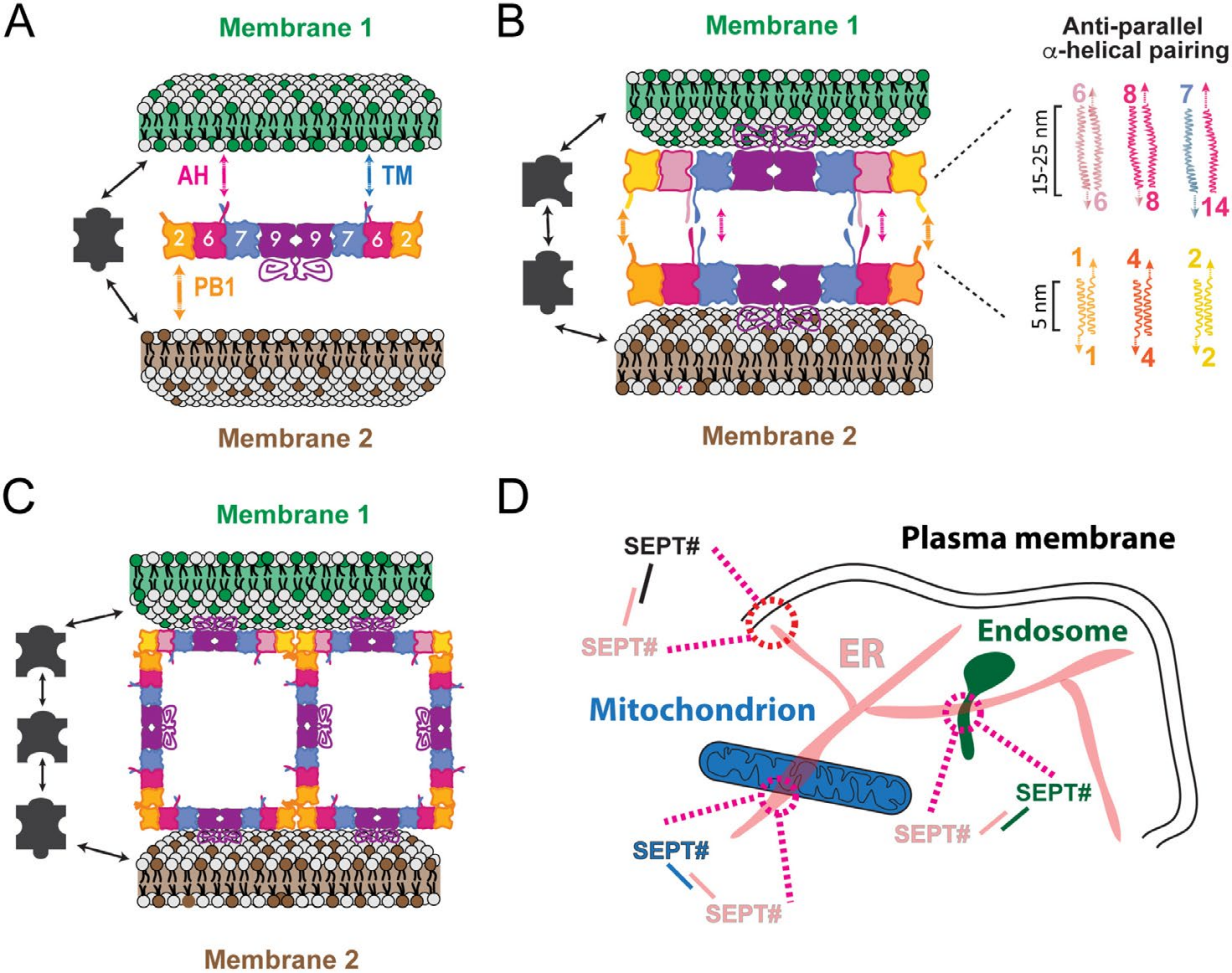
A jigsaw model for septin‐mediated membrane contacts. Analogous to the “knob‐and‐socket” compatibility of jigsaw puzzle pieces, septins may selectively enable or hinder membrane contact sites. (A) Septins bridge two membrane bilayers based on the specificity of their N‐ and C‐terminal lipid‐binding domains, which are located on the ventral and dorsal surfaces of septin polymers, respectively. AH, amphipathic helix; TM, transmembrane domain; PB1, polybasic domain 1. (B) Septin polymers link two apposing membranes by pairing laterally, based on the compatibility of their C‐terminal α‐helical extensions for anti‐parallel interactions. Recent evidence suggests that the C‐terminal coiled‐coil domains of septins have differential preferences for anti‐parallel interactions, as indicated by preferential pairings among septins of the SEPT2, SEPT6, and SEPT7 groups. These interactions may also determine the spacing between two juxtaposed membranes, which could range from 5 to 15–20 nm depending on the length of the C‐terminal extensions of septins belonging to the SEPT6/7 and SEPT2 groups, respectively. (C) Septin filaments may couple septin‐coated membrane bilayers by forming cross‐bridges through orthogonal interactions between opposing septin polymers. The structural and biochemical requirements for this type of interaction are not yet understood; however, orthogonal interactions between septin filaments are prevalent in septin networks with gauze‐like arrangements. (D) Septins may provide a combinatorial code for intracellular membrane contact sites. We hypothesize that membrane organelles bear distinct septin signatures—defined by septin complexes with organelle‐specific paralog and isoform compositions (denoted as SEPT#) — and that membrane contact sites are determined by the compatibility of septin interactions at the interface between membrane organelles. Accordingly, certain septin paralogs and isoforms may promote membrane contact sites at the interface of organelles such as mitochondria and endolysosomes with the ER, as well as between the ER and the plasma membrane (PM).

A major challenge is to determine whether and how septins structurally link two juxtaposing membranes directly, as there is no unequivocal evidence that they do and no established paradigms for how they might do it. We posit that septin filaments possess the structural properties to tether membranes directly, either as individual filaments or alternatively as bundles and/or meshworks of filaments. Individual filaments consist of heteromeric oligomers containing septin paralogs with membrane‐binding domains that extend orthogonally from the linear polymer axis in opposite orientations (Cavini et al. 2021). The first polybasic lipid‐binding domain of septin paralogs from the SEPT2, SEPT3, and SEPT7 groups is positioned at the bottom of the dimeric G‐domain interface, while the membrane‐binding amphipathic helix of SEPT6 group septins extends from the top of G‐interface and is part of an orientationally flexible C‐terminal extension (Sirajuddin et al. 2007; Castro et al. 2020; Cavini et al. 2021; Mendonça et al. 2021) (Figure 1B,C and Figure 5A). This bilateral asymmetry may enable septin filaments to latch onto two opposing membranes through different subunits. Given that septin filaments stack on top of one another on supported lipid bilayers, forming multilayered networks or bundles, it is plausible that the most distally positioned filaments are free to make cis contacts with another lipid bilayer (Jiao et al. 2020; Szuba et al. 2021; Goodchild et al. 2025). Plausibly, these contacts would involve anti‐parallel zippering between the C‐terminal extensions of paralogs of the same septin group in the polymers of the apposed membranes (Leonardo et al. 2021) (Figure 5B). Alternatively, membranes bound to a single layer of septin filaments may become trans‐linked via septins that form orthogonal crossbridges, resembling the rungs of a ladder (Figure 5C). This possibility is supported by in vivo and in vitro observations of septin filament assembly into railroad track or gauze‐like arrangements (Rodal et al. 2005; Bertin et al. 2008; Garcia et al. 2011; Szuba et al. 2021). Although septins are structurally suited for mediating trans interactions between membrane bilayers directly, it remains unknown whether they function as such. Thus, pending further evidence, membrane‐bound septins appear to link adjoining membranes through heterologous proteins, forming heterotypic trans tethers akin to STIM1‐Ora1‐mediated ER‐PM contacts or VAP‐ORLP1‐mediated ER‐late endosome contacts (Voeltz et al. 2024).

If membrane tethers are like the jigsaw puzzle pieces of the organelle connectome, we envisage septins—a family of 13 different paralogs with a multitude of isoforms each—as pieces that selectively promote or hinder MCS. In a jigsaw “knob‐and‐socket” compatibility model, septin paralogs and complexes of distinct compositions associate differentially and selectively with certain membrane organelles and domains, and mediate MCS formation based on their preferential affinity for the proteins or lipids of apposing membranes (Figure 5D). For example, septin complexes that associate with domains of PM curvature owing to subunits (e.g., Sept6) with preferential affinity for the shape and lipids of these domains may selectively interact in trans with septin paralogs enriched on mitochondrial (Sept4) or Golgi (Sept1) membranes (Garcia et al. 2011; Song et al. 2019; Lobato‐Márquez et al. 2023). Thus, septins may provide a code for membrane‐membrane interactions similar to tubulin and septin codes proposed for the spatial regulation of membrane traffic based on selective engagement of motors with specific microtubule tracks (Spiliotis and Kesisova 2021; McKenna et al. 2023).

Septin presence on a subset of microtubules and actin filaments might also be a critical component of septin‐mediated MCS. Recent work revealed that ER and lysosomes associate preferentially with microtubules bearing distinct post‐translational modifications such as polyglutamylation and detyrosination, respectively (Mohan et al. 2019; Zheng et al. 2022), and work from our lab showed that septins preferentially associate with and immobilize multivesicular bodies on a distinct subset of microtubules (Robinson et al. 2024). Thus, septins may enable the formation of microtubule‐ and actin‐based hubs, where clustering of specific organelles catalyzes MCS formation, analogous to how railroad depots facilitate cross‐platform interchanges between different train lines. These hubs can also form at head‐on contacts of cytoskeletal track ends with membrane‐bound septins, as organelles such as the ER associate with microtubule plus ends (Grigoriev et al. 2008; Nourbakhsh et al. 2021), and septins can directly capture microtubule plus ends (Nölke et al. 2016; Nakos et al. 2019).

## 8 Future Directions

In the last two decades, growing research on MCS has upended the long‐held view of membrane organelles as siloed compartments, revealing that organelle connectivity underlies the exchange of signals and metabolites, as well as many cellular processes previously thought to rely solely on cytoplasmic diffusion or vesicular transport (Prinz et al. 2020; Voeltz et al. 2024). As MCS research continues to reshape organelle biology, it is imperative that septins—a major component of cell membranes and the cytoskeleton—be examined through this lens.

In future work, quantitative mapping of the paralogues and isoforms of the septin family to various organelle MCS would be necessary to enhance our understanding of septin selectivity for particular MCS. Upon identification of MCS‐specific septin complex pairs, the next step would be to determine structurally and mechanistically how they link apposing membranes, and how they function in the exchange of signals, metabolites, and ions. Additionally, a key challenge will be to determine the signaling cues and pathways that control septin association with specific organelles and MCS. Post‐translational modifications such as phosphorylation by signaling kinases (Scholz et al. 2015; Yadav et al. 2017; Ageta‐Ishihara, Mizukami, et al. 2025), as well as sumoylation (Ribet et al. 2017), acetylation (Mitchell et al. 2011), and ubiquitylation (Xian et al. 2024) have been found to alter septin assembly and localization patterns, but how these specifically impact the localization and function of septins at MCS remains unknown.

In neurons, ER‐PM contact sites have emerged as essential structures for Ca^2+^ homeostasis and signaling. A body of evidence has cemented septins as integral components and regulators of neuronal SOCE at STIM1‐Orai1 mediated MCS (Deb and Hasan 2016). Whether septins are also present at the periodic ER‐PM junctions of dendrites—where voltage‐gated Ca^2+^ channels coordinate with ryanodine receptors to propagate calcium signals from stimulated dendrite spines—remains unknown (Benedetti et al. 2025). In light of recent findings demonstrating that PM curvature promotes MCS through curvature‐sensing proteins with dedicated motifs (Yang et al. 2024, 2025), it is plausible that septins mediate ER‐PM contacts at sites of their own curvature enrichment, including dendritic spine necks and branch points. At these sites, and more broadly at any septin‐bound MCS, probing for septin‐dependent diffusion barriers may yield valuable mechanistic insights into the exchange of lipids and other metabolites between adjacent membranes, and the structural underpinnings that enable directional delivery without local content mixing and/or fusion.

Going beyond ER‐PM contacts, a better characterization of inter‐organelle contacts is needed to reveal potential associations with septins. While this is more tractable by light microscopy in cell bodies than in the narrow confines of axons and dendrites, where dimensionality severely limits optical resolution, the combination of expansion microscopy and super‐resolution imaging has overcome many of these constraints, enabling visualization that approaches electron microscopy resolution (Gallagher and Zhao 2021; Hümpfer et al. 2024). Electron tomography will be indispensable for resolving how septins are organized at MCS at the ultrastructural level in three dimensions, providing both new insights into current models and a foundation for new mechanistic hypotheses.

In summary, septins are a family of proteins with understudied and underappreciated roles in MCS formation and dynamics. Their selectivity for distinct membrane domains and cytoskeletal tracks, combined with their intrinsic capacity to mediate trans interactions as molecular glues, positions septins as a unique and versatile class of MCS tethers. A deeper understanding of septin function at MCS promises to illuminate fundamental principles of organelle connectivity, and the ability to pharmacologically modulate septin properties—as recently demonstrated for the treatment of Alzheimer's disease (Princen et al. 2024; Hasan 2024) – opens new therapeutic avenues for diseases rooted in MCS dysregulation.

## Author Contributions

**TrishaJean J. Holt:** conceptualization, writing – original draft, writing – review and editing, visualization. **Elias T. Spiliotis:** conceptualization, writing – review and editing, funding acquisition, writing – original draft, visualization, supervision, project administration.

## Funding

This work was supported by the National Institute of General Medical Sciences (5R35GM136337‐07).

## Data Availability

Data sharing not applicable to this article as no datasets were generated or analysed during the current study.
